# Empyema of the Antrum of Highmore

**Published:** 1897-04

**Authors:** Edward E. Gibbons

**Affiliations:** Chief of Clinic to the Professor of Eye and Ear Diseases, Maryland University Hospital, Baltimore.


ARTICLE III.

### EMPYEMA OF THE ANTRUM OF HIGHMORE.

BY EDWARD E. GIBBONS, M. D.,

Chief of Clinic to the Professor of Eye and Ear Diseases, Maryland University Hospital, Baltimore.

On the 22nd of June, 1896, A. S., white male, æt. 19, came to me with the following history: That about one year and a half ago he had suffered with severe pain in and about the left second superior molar tooth. The pain continued two days, accompanied by a considerable swelling of the left side of the face, at which time he consulted a dentist, who finding the tooth carious, drew it, which gave great relief. About one week afterward the patient says he noticed a small, round and very painful swelling beneath the left eye. He immediately consulted his physician, who incised the lump, giving exit to a quantity of pus. From that time until I saw him he had had a sinus below his left eye, the external orifice corresponding to the inferior margin of the orbit, from which pus would gush every time he blew his nose. The sinus, examined with a probe, was found to run backward along the floor of the orbit about one quarter of an inch. The lower eyelid had been engaged in the cicatrix at the mouth of the sinus and pulled down, producing a very unsightly ectropion. The patient had a very decided deflection of his nasal septum to the left side, which interfered with intra-nasal inspection, but, a greatly hypertrophied middle turbinate was seen, occluding the ostium maxillare prohibiting the natural drainage of the antrum through the nose. The history of the case, and my inability to illuminate the eye on the corresponding side, after Davidson's method, as well as the antrum itself, made the diagnosis.

Five days later I perforated the antrum, with a Pope's antrum trephine, through the alveolus of the second molar. The pus which came was very fetid. A soft rubber drainage tube with a flange was introduced, and the end occluded with a stopper to prevent the constant trickling of pus into the patient's mouth, and entrance of food into the cavity. This tube gave the patient absolutely no inconvenience as the flanged end fit snug up to the gum. Examination of the cavity with probe and mirror failed to detect the presence of any necrosed bone or growth.

When the cavity was washed out the solution came freely from the sinus below the orbit. The hypertrophied middle turbinated body was cauterized and the cavity washed out with a solution of permanganate of potash. Two irrigations sufficed to destroy the odor. The cavity was then daily washed out with hydrogen dioxide diluted one-half, and finally in full strength. After about two months the suppuration ceased, and is absent at the present time. The eyelid was released by dividing several bands of connective tissue, by which it was held down, and the ectropion relieved.

A few words about the etiology of these cases. The sinus maxillaris is especially liable to infection from one of two sources; from a diseased tooth, or from the nose. Authorities do not agree as to the most common mode of infection. Kuchenbecker found, out of 31 cases, 33 per cent. the result of dental caries, 22 per cent. of general diseases, 10 per cent. of tumors, 22 per cent. of unknown causes, while only 13 per cent. could be traced to intranasal origin.

The operative treatment of those cases consists in making an opening into the cavity and washing it out daily, until all suppuration has ceased. The older surgeons, says Diffenqach in his "System of Surgery," gave more space to this operation than they did to resection of the jaw. Among the earliest methods of opening the antrum was that of Malinetti, who opened it, in 1675, by making a crucial incision in the cheek and perforating the cavity through the canine fossa. This operation did not find many followers. Our present methods date from the latter part of the last century. About that time Cooper opened the antrum through the mouth, by extracting a tooth and entering through the empty alveolus. If there is a carious molar tooth present, it should be extracted. If the cavity is not opened by this method a strong trocar will easily establish communication. It is far safer, however, to use a drill or trephine. If the teeth are all sound we may then open the sinus through the nose. Hunter opened the cavity through the infundibulum, but drainage was not good, as the point selected was too high up, and there is also great danger of wounding the floor of the orbit. The inferior meatus is the site usually selected for opening the cavity when the trouble is intra-nasal in origin. It can be done with a strong trocar or electric drill. This operation is contra-indicated if decided stenoses of the nasal chambers exist.—*University of Maryland Bulletin.*

